# Dabigatran exhibits low intensity of left atrial spontaneous echo contrast in patients with nonvalvular atrial fibrillation as compared with warfarin

**DOI:** 10.1007/s00380-016-0871-5

**Published:** 2016-07-12

**Authors:** Tetsuya Watanabe, Yukinori Shinoda, Kuniyasu Ikeoka, Hirooki Inui, Hidetada Fukuoka, Akihiro Sunaga, Takashi Kanda, Masaaki Uematsu, Shiro Hoshida

**Affiliations:** 1 Department of Cardiovascular Medicine, Yao Municipal Hospital, 1-3-1 Ryuge-cho, Yao, Osaka, 581-0069 Japan; 2 Cardiovascular Center, Kansai Rosai Hospital, 3-1-69 Inabaso, Amagasaki, Hyogo 660-8511 Japan

**Keywords:** Atrial fibrillation, Transesophageal echocardiography, Spontaneous echo contrast, Dabigatran, Warfarin

## Abstract

The presence of spontaneous echo contrast (SEC) in the left atrium has been reported to be an independent predictor of thromboembolic risk in patients with atrial fibrillation (AF). Dabigatran was associated with lower rates of stroke and systemic embolism as compared with warfarin when administered at a higher dose. Between July 2011 and October 2015, nonvalvular AF patients treated with warfarin or dabigatran who had transesophageal echocardiography prior to ablation therapy for AF were enrolled. The intensity of SEC was classified into four grades, from 0 to 3. Univariate and multivariate analysis was performed to analyze factors associated with SEC. Sixty-five patients were on dabigatran and 65 were on warfarin, with the prothrombin time in therapeutic range. There were no significant differences in the age, CHADS2 score, left atrial dimension, and left atrial appendage flow between the two groups. However, there were more grade 2 or higher patients with left atrial SEC in the warfarin group (*n* = 20) than in the dabigatran group (*n* = 2) (*p* < 0.001). When multivariate regression analysis was performed, grade 2 or higher left atrial SEC was independently associated with no dabigatran usage in addition to high brain natriuretic peptide level and high incidence of diabetes mellitus or persistent AF. Thus, dabigatran exhibited low intensity of left atrial SEC in nonvalvular AF patients as compared with warfarin.

## Introduction

Atrial fibrillation (AF) is the most common arrhythmia, and increases the risk of stroke and systemic embolism [1]. Thromboembolic event rates vary from 2 to 18 % yearly, depending on clinical and/or echocardiographic risk factors [2–5]. In addition to transthoracic echocardiography, transesophageal echocardiography can be a potentially useful diagnostic imaging modality. It can diagnose left atrial thrombi with accuracy, as well as abnormal flow in the left atrium due to stasis of blood flow [6, 7]. This so-called low flow state is characterized by the presence of left atrial spontaneous echo contrast (SEC), and is accompanied by low left atrial appendage peak filling and emptying flow velocities [8–10]. SEC is defined as an echodense whirling pattern that may be visible on transesophageal echocardiogram [11, 12]. SEC is considered to be closely related to thromboembolic events [13, 14]. Hematocrit, fibrinogen concentration, brain natriuretic peptide (BNP), and high-sensitivity C-reactive protein (hs-CRP) have been closely related to SEC [15, 16].

Oral anticoagulation therapy is the standard of care for outpatient prevention of stroke and systemic embolism in patients with AF. Warfarin substantially decreases the risk of stroke in patients with nonvalvular AF, but it increases the risk of major bleeding [2]. The introduction of novel oral anticoagulants (NOACs) provides new options for periprocedural anticoagulation. A direct thrombin inhibitor (DTI), dabigatran was approved as an alternative to warfarin for prevention of stroke and systemic embolism in patients with AF [17]. The intensity of left atrial SEC could be affected by the medication used. However, there are no SEC reports that compare warfarin with dabigatran. The purpose of this study was to examine the difference in the intensity of left atrial SEC between AF patients treated with warfarin and those with dabigatran.

## Materials and methods

### Study patients

This retrospective study included 130 patients who had been admitted for ablation therapy in Yao Municipal Hospital and Kansai Rosai Hospital between July 2011 and October 2015. All of the patients were nonvalvular AF [mean age: 65 ± 9 years; men: 90 (69 %); paroxysmal AF: 56 (43 %)]. The patients were treated with warfarin or dabigatran and underwent transesophageal echocardiography prior to ablation therapy for AF. Oral anticoagulants (warfarin and dabigatran) were interrupted just before ablation theraphy.

### Study design

We essentially adopted the dose of dabigatran similar as the arm of a higher dose in RE-LY study (150 mg twice daily). However, the dose of dabigatran was reduced according to renal dysfunction or concomitant use of other drugs. For the patients with creatinine clearance 30–50 mL/min or concomitant use of P-glycoprotein inhibitors, we reduced dabigatran dose 110 mg twice daily.

Patients with significant mitral valve disease or a valvular prosthesis were excluded from the study. AF was documented from a 12-lead electrocardiogram or Holter electrogram before the transesophageal echocardiography examination. Clinical parameters, such as sex, age, AF type, history of hypertension, heart failure or thromboembolism, and blood tests were analyzed and entered into a regression analysis. A written informed consent was obtained for all patients. The entire investigation conformed to the principles outlined in the Declaration of Helsinki and this study was approved by ethical committee of our hospitals.

### Echocardiographic measurements

Transthoracic and transesophageal echocardiography were performed with a TOSHIBA echocardiogram (ARTIDA™, Toshiba, Tokyo, Japan). Transthoracic echocardiography was performed with a broadband 3-MHz phased-array transducer connected to an ultrasound system and transesophageal echocardiography was performed with a 5-MHz multiplane transducer. Prior to transesophageal echocardiography, all patients fasted for at least 4 h. Premedication was not given, but a local anesthetic was sprayed into the hypopharynx. Transesophageal echocardiography was conducted to evaluate the presence or absence of left atrial thrombus, presence or absence of left atrial SEC, and left atrial appendage emptying peak flow velocity, as described previously [8, 10, 18, 19]. All the patients underwent transthoracic and transesophageal echocardiography after at least 3 weeks on warfarin, with a target prothrombin time-international normalized ratio (PT-INR), or after at least 3 weeks on dabigatran 8 ± 1 h after the last dosage. In the guideline of the Japanese Circulation Society, the suitable level of PT-INR during warfarin therapy for nonvalvular AF is 2.0–3.0 in patients aged <70 years but is 1.6–2.6 in patients aged ≥70 years to avoid bleeding complications.

**Fig. 1:**
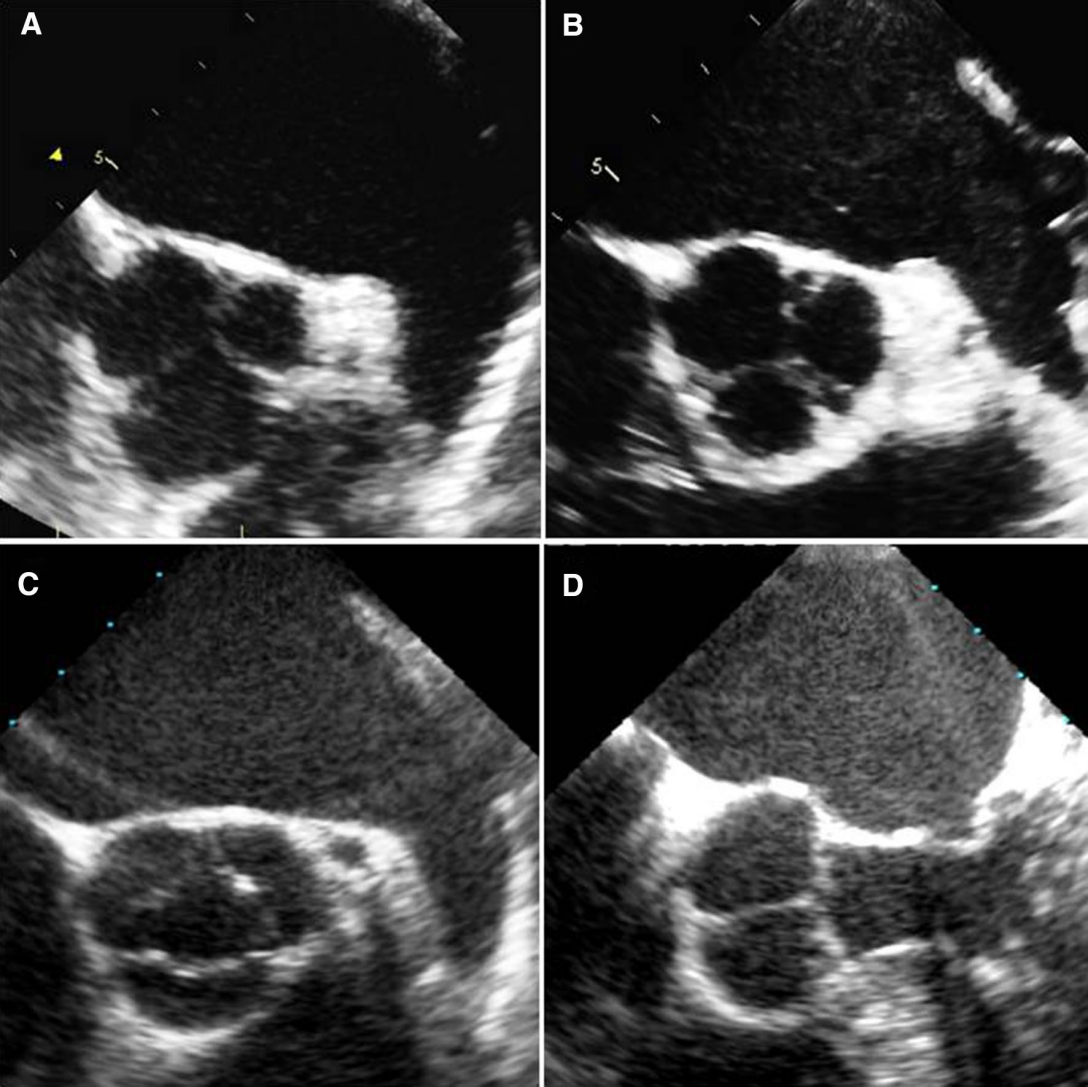
The intensity of left atrial spontaneous echo contrast (SEC) was classified into four grades, from 0 to 3. Grade 0 was no SEC (**a**), grade 1 mild (**b**), grade 2 moderate (**c**) and grade 3 severe (**d**)

The intensity of SEC was classified into four grades, from 0 to 3. Grade 0 was no SEC, and grade 1 was mild, indicating minimal echogenicity in the left atrial appendage. Grade 2 was moderate, and demonstrated a dense swirling pattern in the main cavity. Grade 3 was severe, and demonstrated intense echodensity, with very slow swirling patterns in the main cavity (Fig. 1). For the evaluation of SEC, two experienced observers reviewed the digital images during an offline analysis without current knowledge of the patient and other imaging results.

### Statistical analysis

All statistical analyses were performed using StatFlex for Windows Version 6 (Artech Co. Ltd., Osaka, Japan). Quantitative variables were expressed as mean ± standard deviation. For comparison of measurements between warfarin and dabigatran, Student’s *t* test was used for continuous variables, and the *χ* ^2^ test for categorical variables. Multivariate regression analysis was performed to investigate the independent factors for the presence of moderate/severe left atrial SEC. Values were considered significant when the *p* value was <0.05.

## Results

### Patient backgrounds

Of 130 patients, 65 (50 %) were on warfarin and 65 (50 %) were on dabigatran. In patients treated with dabigatran, the reduced dose (110 mg twice daily) was prescribed in 29 patients. Previous heart failure was present in 27 patients (21 %), hypertension in 75 (58 %), diabetes mellitus in 14 (11 %), and previous cerebral infarction in 9 (7 %). The mean CHADS2 score was 1.1 ± 0.9. Patient backgrounds comparing warfarin and dabigatran groups are presented in Table 1. Age, sex, history of heart failure, hypertension, diabetes mellitus and previous stroke, and CHADS2 score were not different between the two groups. There were lower numbers of people of paroxysmal AF in warfarin group in comparison with the dabigatran group.

**Table 1:** Patient backgrounds comparing warfarin and dabigatran groups

	Warfarin	Dabigatran	*p* value
	(*n* = 65)	(*n* = 65)	
Age (years)	67 ± 8	65 ± 10	*0.113*
Men	46 (71 %)	44 (68 %)	*0.424*
Previous heart failure	15 (23 %)	12 (18 %)	*0.332*
Hypertension	35 (54 %)	40 (62 %)	*0.238*
Diabetes mellitus	5 (8 %)	9 (14 %)	*0.197*
Cerebral infarction	7 (11 %)	2 (3 %)	*0.083*
CHADS2 score	1.2 ± 1.0	1.1 ± 0.9	*0.465*
AF			
Paroxysmal	21 (32 %)	35 (54 %)	*0.011*
Persistent	44 (68 %)	30 (46 %)	

Italics indicate metastatic SNs

*AF* atrial fibrillation

### Comparison between warfarin and dabigatran groups

Laboratory data for warfarin and dabigatran groups are presented in Table 2. Activated partial thromboplastin time (APTT), hematocrit, creatinine clearance, CRP, and BNP were not significantly different between the two groups, but PT-INR was higher in the warfarin group (*p* < 0.001). Table 2 also displays the characteristics of the transthoracic and transesophageal echocardiographic parameters. There were no significant differences in left ventricular end-diastolic dimension, left ventricular end-systolic dimension, left ventricular ejection fraction, left atrial dimension, and left atrial appendage flow between the two groups. However, there were more grade 2 or higher patients with left atrial SEC in the warfarin group compared to the dabigatran group [20/65 (31 %) vs. 2/65 (3 %); *p* < 0.001]. In the two dabigatran-treated patients showing SEC grade 2 or higher, one was treated with a high dose (300 mg/day) and the other with a low dose (220 mg/day). Left atrial thrombus was detected only in five patients treated with warfarin and their mean PT-INR level was 2.1.

**Table 2:** Laboratory and echocardiographic data in warfarin and dabigatran groups

	Warfarin (*n* = 65)	Dabigatran (*n* = 65)	*p* value
Laboratory data			
PT-INR	2.2 ± 0.5	1.2 ± 0.3	<*0.001*
APTT (s)	43 ± 8	44 ± 12	*0.401*
Hematocrit (%)	41 ± 4	41 ± 5	*0.474*
Ccr (mL/min)	67 ± 24	71 ± 21	*0.265*
CRP (mg/dL)	0.14 ± 0.51	0.13 ± 0.37	*0.902*
BNP (pg/mL)	144 ± 143	116 ± 113	*0.226*
Echocardiographic data			
LVDd (mm)	47 ± 5	48 ± 5	*0.418*
LVDs (mm)	31 ± 6	30 ± 4	*0.918*
LVEF (%)	65 ± 8	65 ± 8	*0.910*
LAD (mm)	42 ± 8	41 ± 7	*0.953*
LAA flow (cm/s)	39 ± 20	42 ± 19	*0.394*
Left atrial thrombus	5	0	*0.068*
SEC grade			
Grade 0 or 1 (0/1)	45 (32/13)	63 (44/19)	<*0.001*
Grade 2 or 3 (2/3)	20 (14/6)	2 (1/1)	

Italics indicate metastatic SNs

*PT-INR* prothrombin time-international normalized ratio, *APTT* activated partial thromboplastin time, *Ccr* creatinine clearance, *CRP* C-reactive protein, *BNP* brain natriuretic peptide, *LVDd* left ventricular end-diastolic dimension, *LVDs* left ventricular end-systolic dimension, *LVEF* left ventricular ejection fraction, *LAD* left atrial dimension, *LAA* left atrial appendage, *SEC* spontaneous echo contrast

### Univariate and multivariate analysis for SEC grade

There were significant differences between the patients with SEC grade 0/1 (*n* = 108) and grade 2/3 (*n* = 22) in the incidence of previous heart failure, diabetes mellitus, cerebral infarction and paroxysmal AF, and in CHADS2 score, PT-INR, BNP, left atrial dimension and medication with warfarin or dabigatran (Table 3). When multivariate regression analysis was performed using these significant factors, the patients with SEC grade 2/3 were independently associated with no dabigatran usage [odds ratio: 0.019 (95 % CI 0.001–0.963, *p* = 0.047)]; in addition to high levels of BNP [odds ratio: 1.008 (95 % CI 1.002–1.015, *p* = 0.010)]; and the incidence of diabetes mellitus [odds ratio: 11.30 (95 % CI 1.218–576.6, *p* = 0.018)]; or persistent AF [odds ratio: 0.023 (95 % CI 0.001–0.561, *p* = 0.020)].

**Table 3:** Factors affecting SEC grade

	SEC grade		Univariate	Multivariate	
	0/1 (*n* = 108)	2/3 (*n* = 22)	*p* value	*p* value	Odds ratio (95 % CI)
Age (years)	65 ± 9	69 ± 7	*0.117*	–	–
Men	75 (69 %)	15 (68 %)	*0.554*	–	–
Previous heart failure	19 (18 %)	8 (36 %)	*0.045*	*0.345*	2.599 (0.357–18.90)
Hypertension	60 (56 %)	15 (68 %)	*0.196*	–	–
Diabetes mellitus	8 (7 %)	6 (27 %)	*0.009*	*0.018*	11.30 (1.218–576.6)
Cerebral infarction	5 (5 %)	4 (18 %)	*0.034*	*0.454*	2.900 (0.178–47.26)
Paroxysmal AF	55 (51 %)	1 (5 %)	<*0.001*	*0.020*	0.023 (0.001–0.561)
CHADS2 score	1.0 ± 0.9	1.9 ± 1.0	<*0.001*	*0.240*	1.921 (0.646–5.715)
PT-INR	1.6 ± 0.6	2.2 ± 0.6	<*0.001*	*0.058*	6.175 (0.938–40.64)
APTT (sec)	44 ± 11	44 ± 7	*0.994*	–	–
Hematocrit (%)	41 ± 5	42 ± 5	*0.254*	–	–
Ccr (mL/min)	70 ± 23	64 ± 21	*0.297*	–	–
CRP (mg/dL)	0.11 ± 0.30	0.26 ± 0.88	*0.177*	–	–
BNP (pg/mL)	111 ± 109	223 ± 175	<*0.001*	*0.010*	1.008 (1.002–1.015)
LVDd (mm)	47 ± 5	48 ± 5	*0.757*	–	–
LVDs (mm)	30 ± 5	31 ± 5	*0.388*	–	–
LVEF (%)	65 ± 8	63 ± 10	*0.218*	–	–
LAD (mm)	41 ± 7	44 ± 11	*0.047*	*0.647*	1.018 (0.940–1.103)
Dabigatran usage	63 (58 %)	2 (9 %)	<*0.001*	*0.047*	0.019 (0.001–0.963)

Italics indicate metastatic SNs

*SEC* spontaneous echo contrast, *AF* atrial fibrillation, *PT-INR* prothrombin time-international normalized ratio, *APTT* activated partial thromboplastin time, *Ccr* creatinine clearance, *CRP* C-reactive protein, *BNP* brain natriuretic peptide, *LVDd* left ventricular end-diastolic dimension, *LVDs* left ventricular end-systolic dimension, *LVEF* left ventricular ejection fraction, *LAD* left atrial dimension

## Discussion

Our study showed that there were more grade 2 or higher patients with left atrial SEC in the warfarin group than in the dabigatran group. However, there were no significant differences in age, CHADS2 score, left atrial dimension, and left atrial appendage flow between the warfarin and dabigatran groups. A previous study reported that hematocrit and CRP are independent predictors of SEC in AF patients [20]. In this study, however, these factors were not significantly different between the two groups.

Recently, transesophageal echocardiography has been routinely performed before AF ablation therapy or cardioversion, because the presence or absence of thrombus in the atrium is critical for the ablation or cardioversion procedure. Transesophageal echocardiography is the most sensitive and specific tool to detect thrombus in the left atrium [7, 8, 21]. Transesophageal echocardiography can stratify high-risk patients with nonvalvular AF by identifying left atrial SEC. SEC is a positive predictor of stroke or systemic embolism [22]. Patients with thrombus or moderate/severe SEC in the left atrium must receive intensive anticoagulation therapy to avoid thromboembolic events. However, the intensity of the effects of warfarin and dabigatran on the left atrial SEC may differ.

In recent years, the NOACs, such as dabigatran (DTI), and rivaroxaban, apixaban, and edoxaban (direct Factor Xa inhibitors), have been approved for primary and secondary prevention of stroke in patients with nonvalvular AF. Compared with traditional agents such as warfarin, the NOACs offer benefits in terms of efficacy, safety, and convenience. Dabigatran 150 mg twice daily was the only NOAC with a decreased incidence of ischemic stroke, and without increased risk of bleeding, as compared with warfarin. In RE-LY sub-analysis, there were no treatment-by-region interaction for either dose of dabigatran on stroke, ischemic stroke and death between in Asians and in non-Asians [23]. Moreover, in Asian patients with nonvalvular AF, the magnitude effect for each outcome of dabigatran 220 mg daily was comparable with that of 300 mg dose [24]. In our study, 45 % of dabigatran patients were prescribed 220 mg daily. The main reason of this low dose dabigatran use was due to renal dysfunction or concomitant use of P-glycoprotein inhibitors. Even in these patients, dabigatran 220 mg daily use may be more effective to reduce SEC than warfarin [25].

Dabigatran is a univalent DTI that exerts anticoagulant effects by binding to the active site of thrombin. In a blood clot, the heparin–antithrombin complex cannot bind fibrin-bound thrombin. In contrast, given their mechanism of action, DTIs can bind to and inhibit the activity of not only soluble thrombin but also thrombin bound to fibrin. Since they reduce the thrombin-mediated activation of platelets, DTIs also have an antiplatelet effect. DTIs do not bind to plasma proteins, and therefore, should produce a more predictable response than unfractionated heparin. They should also be more effective than low-molecular-weight heparin because DTIs can inhibit fibrin-bound thrombin [26]. Dabigatran enhances the susceptibility of plasma clots to tissue-plasminogen activator-induced lysis by reducing thrombin-activatable fibrinolysis inhibitor activation, and by altering the clot structure at clinically relevant concentrations [27]. Dabigatran can dissolve a clot by such a mechanism. In fact, the potential thrombolytic effect of dabigatran has been previously reported [28–30].

### Study limitations

This study is associated with some limitations. First, our study population comprised relatively small number of patients recruited from two centers. Therefore, large-number data collected in a multicenter are needed. Second, our study was not performed in a randomized fashion, although the major factors that can affect our primary results were not different between warfarin and dabigatran groups. Dabigatran, a DTI, has a potent antithrombin effect, and in high doses decreased ischemic stroke without increased risk of bleeding in patients with nonvalvular AF, compared with warfarin [16]. Therefore, reduced SEC in the left atrium found in the dabigatran group may be direct evidence for its clinical effect. The finding that moderate or severe left atrial SEC was independently associated with no dabigatran usage confirms this. Finally, in the Japanese guidelines, recommended target PT-INR levels were relatively low compared to other countries in patients aged ≥70 years. This proportion might be affected in our results. A large-scale, randomized, prospective study is needed to confirm the results shown in this study.

## Conclusion

In patients with nonvalvular AF, dabigatran administration is probably more effective for reducing left atrial SEC as compared with warfarin.
